# Effects of *Bacillus amyloliquefaciens* and *Bacillus pumilus* on Rumen and Intestine Morphology and Microbiota in Weanling Jintang Black Goat

**DOI:** 10.3390/ani10091604

**Published:** 2020-09-09

**Authors:** Nanchi Zhang, Li Wang, Yong Wei

**Affiliations:** 1 Key Laboratory of Qinghai-Tibetan Plateau Animal Genetic Resource Reservation and Utilization, Ministry of Education and Sichuan Province, Southwest Minzu University, Chengdu 610041, China; zhangnanchi@126.com; 2 Animal Genetics and Breeding Key Laboratory of Sichuan Province, Animal Science Academy of Sichuan Province, Chengdu 610066, China

**Keywords:** *Bacillus amyloliquefaciens*, *Bacillus pumilus*, Jintang black goat, ruminal microbiota, cecal microbiota

## Abstract

**Simple Summary:**

Selecting *Bacillus amyloliquefaciens* and *Bacillus pumilus* in the present work, antibiotic substitutes were found for weaned black goat health and growth. This study indicated that *Bacillus amyloliquefaciens* fsznc-06 and *Bacillus pumilus* fsznc-09 improved the development of rumen and small intestine in weanling Jintang black goats. They also regulated microbial communities by increasing the richness of beneficial bacteria, but decreasing that of pathogenic bacteria in rumen and caecum for nutrient metabolism and promoting healthy conditions.

**Abstract:**

The importance of *Bacillus* as feed additives in animals’ production is well recognized. *Bacillus amyloliquefaciens* and *Bacillus pumilus* are involved in promoting animal growth performance and immunological indicators. However, their precise roles in the modulation of microbiota and immune response in goat rumen and intestines have not been investigated. The aim of the current work was to evaluate the impacts of *Bacillus amyloliquefaciens* fsznc-06 and *Bacillus pumilus* fsznc-09 in the development of rumen and small intestinal and microbial communities in rumen and caecum of weanling Jintang black goats. Morphological alterations of rumen and small intestine (duodenum, jejunum, and ileum) were evaluated by histochemical staining, and ruminal contents and cecal feces were analyzed by 16S rRNA sequencing in an Illumina NovaSeq platform. Morphological analysis showed that feeding weanling goats with *Bacillus amyloliquefaciens* fsznc-06 or *Bacillus pumilus* fsznc-09 enhanced ruminal papilla and small intestinal villus growth. In addition, 16S rRNA sequencing analysis indicated that microbial richness and diversity (Shannon, Simpson, Chao1, and ACE) and the relative richness of multiple or potential beneficial bacteria were higher in weaned black goats fed on *Bacillus amyloliquefaciens* fsznc-06 or *Bacillus pumilus* fsznc-09, but that of multiple or potentially pathogenic bacteria were lower, as compared with the control group. Tax4Fun analysis predicting the functional profiling of microbial communities showed that microbial communities in rumen or caecum were highly influential on metabolism and organism systems after feeding weanling goats with *Bacillus amyloliquefaciens* fsznc-06 or *Bacillus pumilus* fsznc-09. It was suggested that *Bacillus amyloliquefaciens* fsznc-06 and *Bacillus pumilus* fsznc-09 might be an auspicious antibiotic alternative to improve black goat growth and health by changing rumen and gut microbiota positively.

## 1. Introduction

The key element of livestock farming is feed, which has been gaining specific attention to improving animal growth performance. Growth-promoting antibiotics possess extensive utilization in improving animal growth and preventing post-weaning diarrhea in goats. Nevertheless, using antibiotics routinely is questionable in present society due to the increasing antibiotic resistance of intestinal microbes to antibacterial drugs, which may enhance the potential pathogenic bacteria population [1,2]. Natural and safe alternatives with similar activities and potential to improve animal growth and immunity, and maintain bio-safety are in urgent need of development. It is well accepted that probiotics are better alternatives. *Bacillus* have been gaining attention in recent years as a growth-promoting antibiotics alternative due to their animal health benefits and the potential to survive the harsh manufacturing conditions in animal feed production. Therefore, the use of *Bacillus* as feed additives rather than antibiotics is justified in current research. An increasing number of *Bacillus* strains have been developed for use as animal feed additives [3,4].

Previous studies have reported that *Bacillus* improves animal growth performance, immune function, and intestinal development, and it regulates intestinal microbial communities [5,6]. Many reports have demonstrated that *Bacillus amyloliquefaciens* and *Bacillus pumilus* as feed additives have significant functions for resisting oxidation and inflammatory processes, promoting growth performance and immunological indicators of animals, and stimulating the beneficial bacteria proliferation and preventing the harmful bacteria reproduction. In this regard, *Bacillus amyloliquefaciens* SC06 was shown to regulate antioxidant potential and host gut microbiota [7]. Truong et al. reported that a mixture of *Bacillus amyloliquefaciens* and *Bacillus pumilus* significantly increased the average weight gain (AWG) of *Pangasianodon hypophthalmus* [8]. Furthermore, Prieto et al. showed that *Bacillus pumilus* WIT588 treatment decreased ileal *Escherichia coli* counts similarly to medication [9]. These effects may be due to *Bacillus* performance in intestinal microbiota, immunity, gene expression, intestinal morphology, among others [6,10].

We hypothesized that *Bacillus amyloliquefaciens* and *Bacillus pumilus* regulate microbiota and improve growth performance and immunity in rumen and intestine of black goats, which has not been investigated. In this study, *Bacillus amyloliquefaciens* fsznc-06 and *Bacillus pumilus* fsznc-09 were used as feed additives for Jintang black goats to evaluate some relevant biological functions, whose findings will benefit animal husbandry.

## 2. Materials and Methods

### 2.1. Animal Experimental Design

The liquid probiotics’ beneficial roles on rumen and intestines were assessed in weanling of 30 male Jintang black goats (80-day-old), which were raised on a usual farm (Chengdu, Sichuan, China). They were randomly distributed in BA-treated (*Bacillus amyloliquefaciens* treated group), BP-treated (*Bacillus pumilus* treated group), and control groups (10 goats per group and individually housed). The 30-day experiment was developed after 1 week of adaptation. Water and food were given *ad libitum* during the entire experimental period. The BA-treated group was fed with 1 mL of *Bacillus amyloliquefaciens* fsznc-06 at a concentration of 10^9^ CFU/mL (CFU, colony unit forming) every 2 days; the BP-treated group was fed with 1 mL of *Bacillus pumilus* fsznc-09 at a concentration of 10^9^ CFU/mL every 2 days [6], whereas the control group was fed with 1 mL of 0.9% stroke-physiological saline solution every 2 days. Liquid probiotics or stroke-physiological saline solution were orally fed to young goats. *Bacillus amyloliquefaciens* fsznc-06 (GenBank: MN159063) and *Bacillus pumilus* fsznc-09 (GenBank: MN334334) were provided by the College of Life Science and Technology, Southwest Minzu University (Chengdu, Sichuan, China). The Animal Ethic Committee of Southwest Minzu University, Chengdu, Sichuan, China approved all experiments while meeting all ethical standards. Table 1 represents the nutrient composition and dietary ingredients.

### 2.2. Effect of Bacillus amyloliquefaciens and Bacillus pumilus on Rumen and Small Intestine Morphology

At the end of the animal study, goats were sacrificed for sampling. Then, the rumen and small intestine were collected and fixed with 4% formaldehyde, rooted in paraffin, and marked with eosin and hematoxylin, as previously reported [11]. Images of small intestine samples were observed under electron microscopy (BA400Digital; Motic China Group Co., LTD., Xiamen, Fujian, China). Papillae length, papillae width, epithelium thickness of rumen, villus length, crypt depth, and villus width of the small intestine were evaluated with Image-Pro Plus 6.0 software (Media Cybernetics, Inc., Rockville, MD, USA).

### 2.3. Gut Microbiota Analyses

The goat rumen content and caecum feces samples were collected using sterile tubes, immediately frozen in dry ice and kept at −80 °C for the consequent fecal microbiota analyses. V3–V4 areas of bacterial 16S ribosomal RNA gene were investigated for microbial diversity. Ion Plus Fragment Library Kit 48 rxns (Thermo Fisher, Rockford, IL, USA) was utilized for libraries construction and lon S5TMXL (Thermo Fisher, Rockford, IL, USA) was utilized for sequencing. All fecal samples were analyzed by Novogene Co. LTD (Beijing, China). Details on rumen and caecum microbiota analyses are provided in File S1 (Provided by Novogene Co. LTD).

### 2.4. Statistical Analysis

SPSS 26.0 (IBM Corp., Chicago, IL, USA) was used to perform statistical analyses. The data were assessed using one-way ANOVA, the Tukey’s-b test method was used to perform the comparative analysis. Data represent mean ± SD.

## 3. Results

### 3.1. Effect of *Bacillus amyloliquefaciens* and *Bacillus pumilus* on Rumen and Small Intestine Morphology

**Figure 1:**
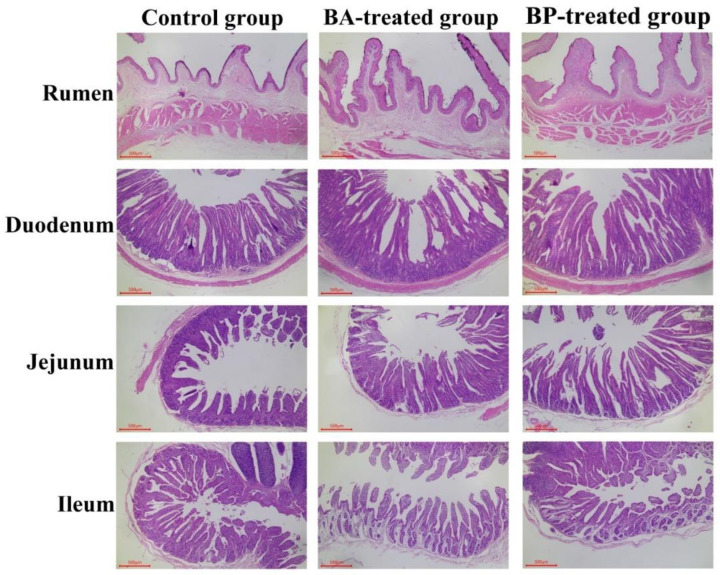
Cross-section of hematoxylin and eosin marking the rumen and small intestine.

The results of histochemical staining in rumen and small intestine are shown in Figure 1 and Table 2. The papilla length and papilla width in the rumen of the BA-treated group and BP-treated group considerably reduced (*p* < 0.05), and the epithelium thickness in the rumen of the BP-treated group increased significantly (*p* < 0.05) in comparison to the control group. The villus height in duodenum and jejunum, the villus width in ileum, and the ratio of villus height to crypt depth (V/C) in duodenum, jejunum, and ileum of the BA-treated group and BP-treated group considerably incremented (*p* < 0.01), and the villus width in the jejunum of the BP-treated group considerably incremented (*p* < 0.01) compared to the control group. Furthermore, the crypt depth in the ileum and duodenum of the BA-treated group and BP-treated group significantly decreased (*p* < 0.01), but the crypt depth in the jejunum of the BA-treated group and BP-treated group incremented significantly (*p* < 0.01) in comparison to the control group.

### 3.2. Analyzing the Bacterial Community Structure by 16S rRNA Sequencing

**Figure 2:**
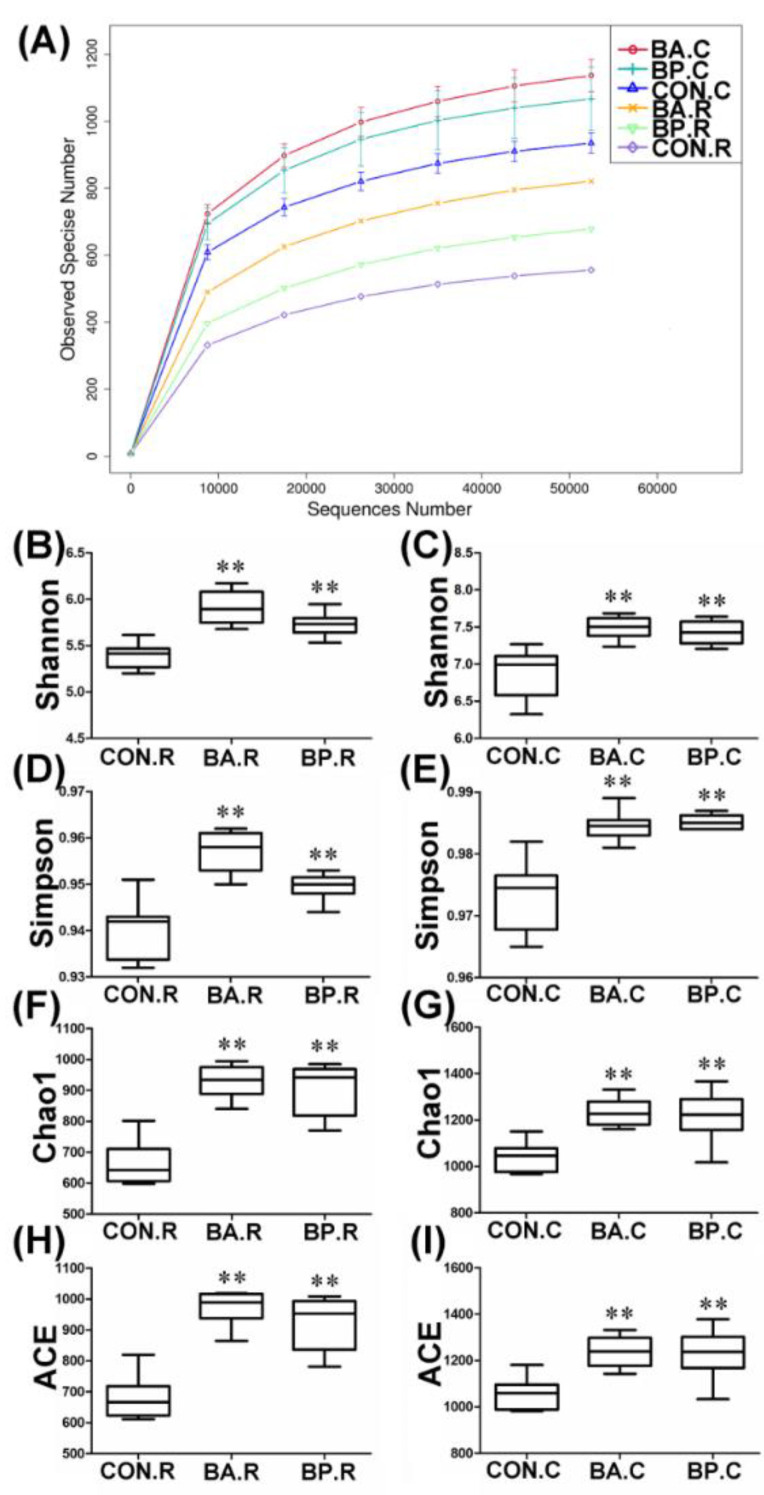
Rarefaction curve and boxplot of differences in bacterial community richness and diversity. (**A**) Rarefaction curve, (**B**,**C**) Shannon index, (**D**,**E**) Simpson index, (**F**,**G**) Chao1 index, and (**H**,**I**) ACE (Abundance-based Coverage Estimator) index. BA.R, BP.R, and CON.R indicate rumen content samples of BA-treated, BP-treated, and control groups, respectively; BA.C, BP.C, and CON.C indicate caecum feces samples of BA-treated, BP-treated, and control groups, respectively. ** *p* < 0.01, compared with the control group.

The V3–V4 area of 16S rRNA gene was sequenced respectively from 30 rumen content samples and 30 caecum feces samples via the Illumina NovaSeq high-throughput sequencing platform. Followed by eliminating chimeric and incorrect sequences, 5,503,599 effective tags were selected from 5,599,842 tags. For every specimen, the combined sequences were within 95.93–99.35%. With an average length of 415 bp, 91,727 sequences were obtained per sample, in average. Based on the rarefaction curves, the sampling depth was sufficient for assessing the bacterial communities (Figure 2A). The criterion of 97% sequence similarity at the species level was used to identify a total of 2056 OTUs (Operational Taxonomic Units) in rumen content samples and 4238 OTUs in caecum feces samples. Finally, 685 OTUs (Good’s coverage) per rumen content samples and 1413 OTUs per caecum feces samples were identified. The determined microbial diversity indices and sequence information of the rumen content specimens and caecum feces samples are shown in Figure 2B–I. The Shannon, Simpson, Chao1, and ACE (Abundance-based Coverage Estimator) indexes in the BA-treated group and BP-treated group were higher compared with the control group (*p* < 0.01), indicating that probiotics increased microbial diversity and richness in black goat ruminal contents and cecal feces.

### 3.3. Diversity and Compositions of Microbiota

**Figure 3:**
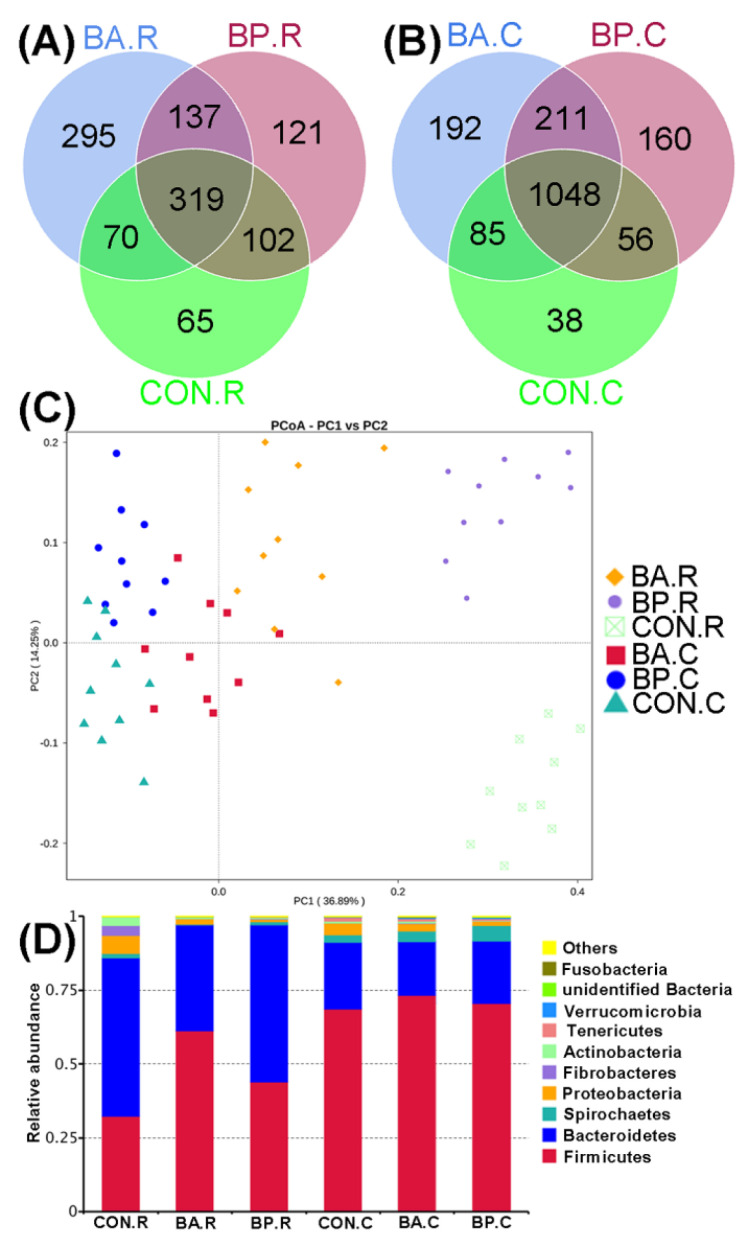
Venn and Principal Coordinates Analysis for microbial composition. (**A**,**B**) Venn diagrams for bacterial OTUs (Operational Taxonomic Units), (**C**) Principal coordinate analysis in terms of unweighted UniFrac metrics, and (**D**) Composition of microbial communities calculated at the phylum level. BA.R, BP.R, and CON.R indicate rumen content samples of BA-treated, BP-treated, and control groups, respectively; BA.C, BP.C, and CON.C indicate caecum feces samples of BA-treated, BP-treated, and control groups, respectively.

As shown in Figure 3A,B, the overall number of OTUs in ruminal contents and cecal feces in BA and BP groups were higher than those of the control group, respectively. The microbial communities in the rumen and caecum of the control group possess the least number of exclusive OTUs amongst the three groups. Based on the Principal Coordinates Analysis (PCoA) of bacterial OTUs in terms of the un-weighted UniFrac distance metrics, there were considerable differences in the compositions of microbial communities in the caecum and rumen among the three groups (Figure 3C). Caecum feces samples were closely clustered, whereas rumen content samples were loosely clustered, indicating that the influence of probiotics on rumen microbial communities was higher than that on caecum microbial communities. At the phylum levels (Figure 3D), Firmicutes was the dominant bacteria with the highest relative abundances, which was higher in the rumen and caecum of BA and BP groups than in the control group, respectively. Nevertheless, the relative abundance of Bacteroidetes in the rumen and caecum of the BA group was lower compared to the control and BP groups.

**Figure 4:**
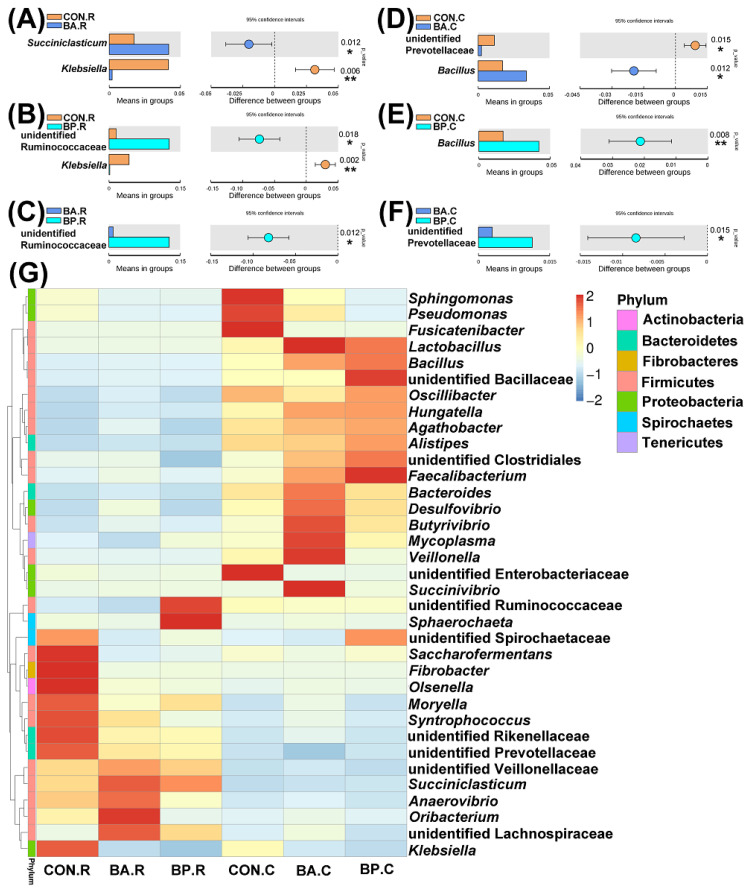
Analyses and heatmap of microbial community. (**A**–**F**) Differences in microbial community at the genera levels by the Student’s t-test. (**G**) Microbial community heatmap at the genera levels. BA.R, BP.R, and CON.R indicate rumen content samples of BA-treated, BP-treated, and control groups, respectively; BA.C, BP.C, and CON.C indicate caecum feces samples of BA-treated, BP-treated, and control groups, respectively. * *p* < 0.05, ** *p* < 0.01.

As observed in Figure 4A–C, the relative richness of *Succiniclasticum* in the BA group rumen and unidentified Ruminococcaceae in the BP group rumen increased significantly, in comparison to the control group (*p* < 0.05). The relative abundance of *Klebsiella* in the rumen of the BA and BP groups decreased significantly in comparison to the control group (*p* < 0.01). According to Figure 4D–F, the relative abundance of *Bacillus* in the caecum of the BA and BP groups increased significantly in comparison to the control group (*p* < 0.05). The relative abundance of unidentified Prevotellaceae in the caecum of the BA group decreased reduced considerably in comparison to the control group (*p* < 0.05). Furthermore, the relative abundance of unidentified Prevotellaceae in the caecum of the BP group increased significantly in comparison to the BA group (*p* < 0.05). The abundances of the top 35 microbial communities in the rumen and caecum at the genus level were provided in the heatmap clustered hierarchically (Figure 4G). The relative richness of *Klebsiella* in the BA and BP group rumens was lower than those in the control group, and the relative abundance of *Succiniclasticum* and unidentified Lachnospiraceae in the BA and BP group rumens were higher compared to the control group. In addition, the relative abundance of *Pseudomonas* in the BA and BP group caecums was lower compared to the control group, and the relative abundance of *Lactobacillus* and *Bacillus* in the BA and BP group caecums was greater compared to the control group.

Network analysis at the genus level indicated that the level of connectivity within the microbiota in the rumen and caecum of goats was high, resulting in the identification of 66 nodes and 148 interactions (Figure S1). The correlation linkages of *Oscillibacter*, *Moryella*, *Desulfobulbus,* and *Negativibacillus* with other bacteria were most complex, respectively. In addition, *Bacillus* positively correlated with *Hungatella*, *Faecalibacterium*, *Intestinimonas*, *Fournierella*, *Ruminiclostridium*, *Blautia*, *Roseburia,* and *Caproiciproducens*.

### 3.4. Differences in Microbial Communities

**Figure 5:**
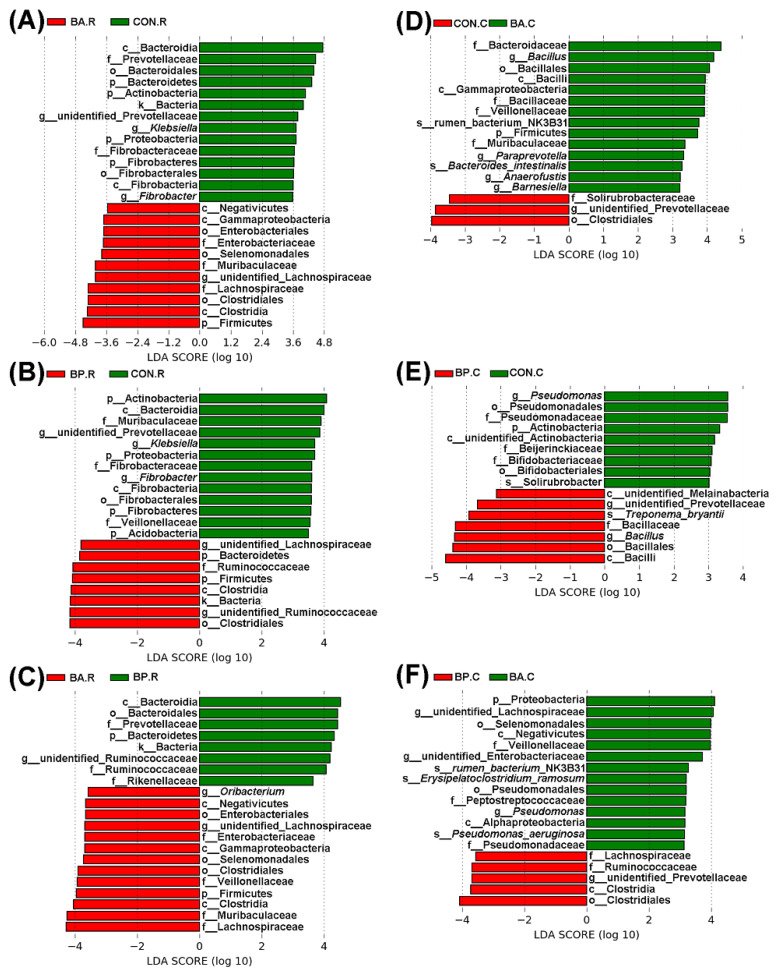
The LDA (Linear Discriminant Analysis) at the genus level. Utilizing linear discriminant analysis coupled with effect size (LEfSe), bacterial taxa at the genus level were identified significantly through the default parameters between the control and BA groups (**A**,**D**), between the control and BP groups (**B**,**E**), and between the BA and BP groups (**C**,**F**), respectively. Only the genera with a significant linear discriminant analysis threshold >3.5 (**A**–**C**) or >3 (**D**–**F**) are shown. BA.R, BP.R, and CON.R indicate rumen content samples of BA-treated, BP-treated, and control groups, respectively; BA.C, BP.C, and CON.C indicate caecum feces samples of BA-treated, BP-treated, and control groups, respectively.

A comparison was made for the OTUs of each group to recognize the significant abundant bacterial taxa against various treatments. Moreover, the biomarkers were detected using LEfSe with a 3 or 3.5-threshold value of Linear Discriminant Analysis (LDA) at the genus level. In rumen microbial communities (3.5 threshold value), there were 14 taxa in the control group and 11 taxa in the BA group (Figure 5A), 13 taxa in the control group and 8 taxa in the BP group (Figure 5B), and 13 taxa in the BA group and 8 taxa in the BP group (Figure 5C). In caecum microbial communities (3 threshold value), there were 3 taxa in the control group and 14 taxa in the BA group (Figure 5D), 9 taxa in the control group and 7 taxa in the BP group (Figure 5E), and 14 taxa in the BA group and 5 taxa in the BP group (Figure 5F).

### 3.5. Estimated Functional Profiles of Microbial Communities

**Figure 6:**
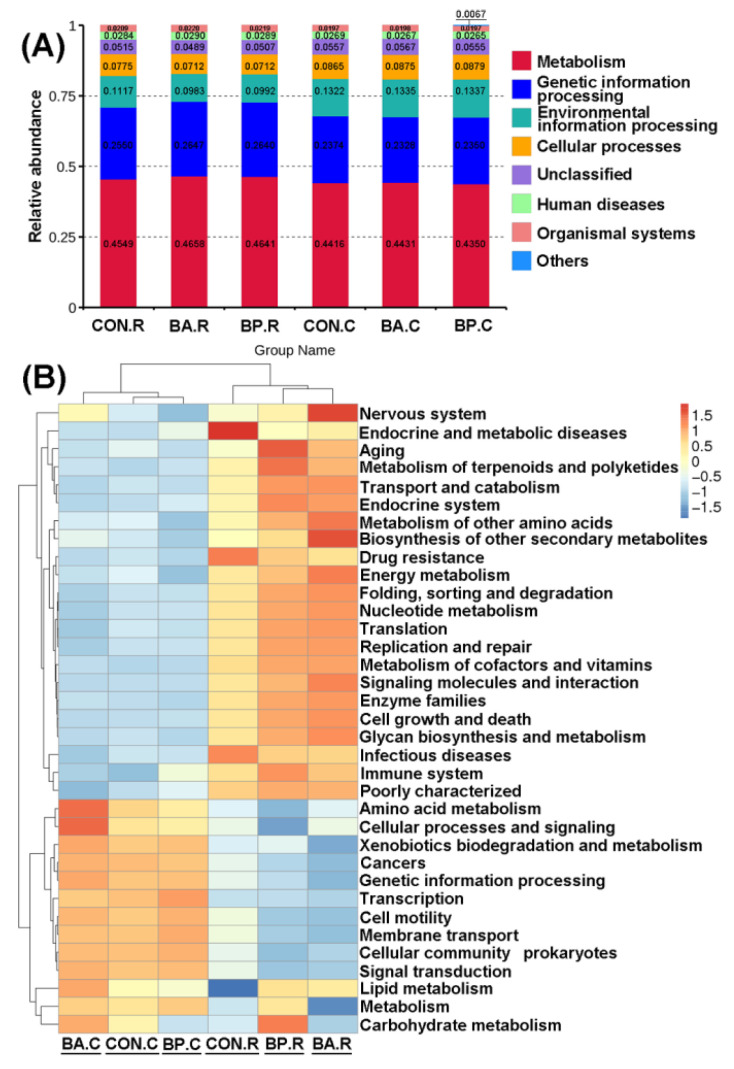
Estimation for Kyoto Encyclopedia of Genes and Genomes (KEGG) paths of microbial communities by the Tax4Fun program. (**A**) KEGG pathway of microbial communities at the level 1 and (**B**) Heatmap of predicted functions of microbial communities at KEGG level 2. BA.R, BP.R, and CON.R indicate rumen content samples of BA-treated, BP-treated, and control groups, respectively. BA.C, BP.C, and CON.C indicate caecum feces samples of BA-treated, BP-treated, and control groups, respectively.

For predicting the potential functional profiles of microbial communities of rumen and caecum in nutrient metabolism for weanling Jintang black goats fed with *Bacillus amyloliquefaciens* fsznc-06 and *Bacillus pumilus* fsznc-09, Kyoto Encyclopedia of Genes and Genomes (KEGG) paths were assessed by Tax4Fun program. At KEGG level 1, the biological pathways in microbial communities of rumen and caecum mainly included metabolism, environmental information processing, genetic information processing, human diseases, cellular processes, and organism systems (Figure 6A). The relative abundance of metabolism and genetic information processing were the highest in each group. The abundances of the top 35 KEGG pathways in rumen and caecum at the KEGG level 2 were provided in the hierarchically clustered heatmap (Figure 6B). In the functional pathways of rumen microbial communities, the relative abundance of the nervous system, biosynthesizing of other secondary metabolites, and lipid metabolism in the BA group and aging, metabolism of polyketides and terpenoids, and carbohydrate metabolism in the BP group were all higher compared to the control group, whereas the relative abundance of endocrine and metabolic diseases, drug resistance, and infectious diseases in the BA and BP groups were lower compared to the control group. In functional pathways of caecum microbial communities, the relative abundance of the nervous system, amino acid metabolism, cellular processes, and signaling in the BA group and the immune system in the BP group were higher compared to the control group, whereas the relative abundance of carbohydrate metabolism in the BP group was lower compared to the control group.

## 4. Discussion

Feed antibiotics have been extensively utilized for preventing weaning stress since the early 1950s in animal husbandry for promoting growth and preventing infections [12]. Nevertheless, antibiotics will be prescribed all over the world owing to their side effects, including antimicrobial resistance and residues in animal products. Therefore, replacing antibiotics with *Bacillus* as feed additives becomes an issue of current research interest. The papilla and villus are key elements of the digestive tract included in absorbing nutrients into the body system across the rumen and small intestines [13,14]. The role and influence of rumen and small intestine in digestion is closely associated with their mucosal structure, particularly the shape and size of ruminal papilla and small intestinal villus [15]. Our results indicated that feeding weanling goats with *Bacillus amyloliquefaciens* fsznc-06 or *Bacillus pumilus* fsznc-09 produced a significant effect on the growth of ruminal papilla and small intestinal villus, which is in agreement with a previous study reporting that feeding with *Bacillus amyloliquefaciens* C-1 for 30 d increased rumen development and improved intestinal in growth-retarded animals [16].

Many microorganisms inhabit the rumen and intestines of ruminants. Adequate nutrients and a stable space environment are provided for the host for the microbiota in rumen and intestines, and the microorganisms contribute to nutrient absorption and digestion, as well as regulation of the host physiological functions. Recently, by developing the sequencing technology, the Illumina sequencing platform was utilized for the successful detection of the diversity of many animals digestive tract microbiota, including piglet, goat, and cow [17,18,19]. However, the precise roles of *Bacillus* in modulating the immune and microbiota response in goat rumen and intestines remain to be elucidated. Therefore, we identified the effect of probiotics in rumen and caecum microbial communities by 16S rRNA sequencing analysis. Numerous studies have demonstrated that *Bacillus* strains not only possess broad-spectrum antibacterial activity and promote the beneficial bacteria proliferation in animal rumen and intestines, but they also have adequate safety and prebiotic effects [20,21,22]. The high diversity indexes, including Shannon, Simpson, Chao1, and ACE (Abundance-based Coverage Estimator) indicated that *Bacillus amyloliquefaciens* fsznc-06 or *Bacillus pumilus* fsznc-09 treatment increased the richness of microbiota in rumen and caecum, compared with the control group. A good digestive tract microbiota benefits the host by regulation of the physiological procedure and mucosal immunity, from producing antimicrobial substances to suppressing the proliferation of digestive tract pathogens [23], leading to a highly efficient rumen function [24] and improving growth performance through improving the digestive tract microbial ecosystem [25]. In the present study, Firmicutes and Bacteroidetes were the two dominant phyla in the rumen and caecum, which is in agreement with previous studies [26,27]. Our research indicated that the Firmicutes abundance in the rumen and caecum increased after feeding weanling goats with *Bacillus amyloliquefaciens* fsznc-06 or *Bacillus pumilus* fsznc-09, but the Bacteroidetes abundance decreased in rumen and caecum, indicating that *Bacillus amyloliquefaciens* fsznc-06 and *Bacillus pumilus* fsznc-09 improved Firmicutes proliferation. The relative abundances of multiple or potential beneficial bacteria were higher at the genus level in the BA and BP groups compared to the control group, including *Lactobacillus*, *Bacillus*, *Succiniclasticum*, unidentified Lachnospiraceae, among others; however, the relative abundances of multiple or potentially pathogenic bacteria were lower in the BA and BP groups compared to the control group, including *Pseudomonas*, unidentified Enterobacteriaceae, and *Klebsiella*, among others. These results indicated that feeding weanling goats with *Bacillus amyloliquefaciens* fsznc-06 or *Bacillus pumilus* fsznc-09 promoted the growth of probiotics and inhibited pathogenic bacteria growth in the digestive tract, which agrees with a previous report related to feeding broiler chickens and grouper (*Epinephelus coioides*) with *Bacillus amyloliquefaciens* TL and *Bacillus pumilus* SE5 respectively [10,28]. It is well accepted that there is an interaction between microbial communities in rumen and caecum. Hence, a correlation network analysis was performed in the current work to discover these connections well. Our results indicated that these correlations were extraordinarily complex and inter-based. *Bacillus* was directly or indirectly positively correlated with multiple beneficial bacteria, such as *Faecalibacterium* and *Roseburia*, among others. *Faecalibacterium* is one of the butyrate-producing bacteria of the colon, which is considered an important pro-inflammatory substance, and its abundance in the gut negatively correlates with obesity [29,30]. It has been also observed that *Roseburia* protects colon epithelial cells from inflammatory damage [31].

Against the increased community diversity and richness and the changed microbial composition, the potential functions of microbial communities in the rumen and caecum of probiotics-treated goats differed from those in the control group, as indicated by the Tax4Fun analysis. Our results showed that the microbial communities in rumen or caecum had an enhanced potential to influence metabolism (amino acid metabolism, biosynthesis of other secondary metabolites, energy metabolism, among others) and organism systems (nervous system, aging, among others) after feeding weanling goats with *Bacillus amyloliquefaciens* fsznc-06; the rumen microbial communities had an enhanced effect in influencing the metabolism (metabolism of terpenoids and polyketides, lipid metabolism, carbohydrate metabolism, etc.) and organism systems (immune system, endocrine system, aging, etc.) after feeding weanling goats with *Bacillus pumilus* fsznc-09; however, the microbial communities in rumen had a weakened capacity to influence human diseases (infectious diseases, endocrine and metabolic diseases, drug resistance) after feeding weanling goats with *Bacillus amyloliquefaciens* fsznc-06 or *Bacillus pumilus* fsznc-09.

## 5. Conclusions

This study showed that *Bacillus amyloliquefaciens* fsznc-06 and *Bacillus pumilus* fsznc-09 improved the development of rumen and small intestines in weanling Jintang black goats. They also regulated microbial communities by increasing the richness of beneficial bacteria, but decreasing that of pathogenic bacteria in rumen and caecum, for nutrient metabolism and promoting healthy conditions.

## Figures and Tables

**Table 1:** The composition and ingredients in the experimental diets.

Items	Diet Level
Ingredients	%
Corn	47.20
Soybean meal	19.00
Chinese wildrye grass hay	15.00
Wheat bran	7.60
Corn germ meal	7.50
Limestone	1.40
Calcium hydrogen phosphate	0.45
NaCl	0.45
L-Lysine	0.40
Premix *	1.00
Analyzed composition (% of dry matter)	
Metabolizable energy (MJ/kg)	10.37
Crude protein	14.63
Ether extract	3.53
Crude ash	6.45
Neutral detergent fiber	23.69
Acid detergent fiber	8.23
Lysine	0.50
Calcium	1.04
Phosphorus	0.45

* Premix composition per kg diet: 15000 IU vitamin A, 5000 IU vitamin D_3_, 55 IU vitamin E, 60 mg FeSO_4_·H_2_O, 50 mg MnSO_4_·H_2_O, 12 mg CuSO_4_·5H_2_O, 90 mg ZnSO_4_·H_2_O, 0.35 mg NaSeO_3_, 1 mg KIO_3_, 0.36 mg CoCl_2_·6H_2_O.

**Table 2:** Effects of probiotics on the black goat rumen and small intestine.

Items	Groups			SEM	*p*-Value
	CON	BA	BP		
Rumen					
Papilla length (mm)	2.68 ± 0.29 ^C^	3.48 ± 0.43 ^B^	4.65 ± 0.33 ^A^	0.163	<0.001
Papilla width (mm)	1.07 ± 0.09 ^Bb^	1.25 ± 0.12 ^Ba^	1.78 ± 0.23 ^A^	0.626	<0.001
Epithelium thickness (μm)	89.32 ± 5.99^Ab^	92.03 ± 2.49 ^Aab^	96.08 ± 7.34 ^Aa^	1.121	0.040
Duodenum					
Villus height (μm)	915.38 ± 56.83 ^B^	1041.17 ± 32.05 ^A^	1078.12 ± 26.92 ^A^	14.794	<0.001
Crypt depth (μm)	414.94 ± 9.06 ^A^	326.01 ± 7.44 ^C^	360.09 ± 6.01 ^B^	6.933	<0.001
V/C	2.21 ± 0.17 ^C^	3.19 ± 0.12 ^A^	2.99 ± 0.08 ^B^	0.082	<0.001
Villus width (μm)	92.17 ± 6.85	91.11 ± 9.66	92.56 ± 14.75	1.927	0.954
Jejunum					
Villus height (μm)	424.23 ± 37.77 ^C^	562.80 ± 35.28 ^B^	818.90 ± 43.14 ^A^	31.122	<0.001
Crypt depth (μm)	214.22 ± 8.11 ^C^	226.14 ± 7.21 ^B^	301.44 ± 6.61 ^A^	7.286	<0.001
V/C	1.98 ± 0.16 ^C^	2.49 ± 0.17 ^B^	2.72 ± 0.13 ^A^	0.064	<0.001
Villus width (μm)	97.61 ± 17.17 ^Bb^	115.72 ± 20.17 ^ABb^	143.31 ± 28.03 ^Aa^	5.250	<0.001
Ileum					
Villus height (μm)	517.30 ± 44.73	558.07 ± 68.07	540.69 ± 36.97	9.612	0.227
Crypt depth (μm)	299.81 ± 10.46 ^A^	248.31 ± 10.77 ^B^	218.98 ± 7.09 ^C^	6.430	<0.001
V/C	1.73 ± 0.17 ^Bc^	2.25 ± 0.26 ^Ab^	2.47 ± 0.19 ^Aa^	0.069	<0.001
Villus width (μm)	102.64 ± 13.31 ^Bb^	132.31 ± 23.99 ^Aa^	141.10 ± 26.07 ^Aa^	4.915	0.001

Note: Data represent mean ± SD (*N* = 10). In the same row, values with various capital letter superscripts indicate considerable difference (*p* < 0.01), whereas different small letter superscripts indicate significant difference (*p* < 0.05), and the same small letter superscripts or without letter indicates no considerable difference (*p* > 0.05). V/C, the villus height-crypt depth ratio; CON, control group; BA, BA-treated group (*Bacillus amyloliquefaciens treated group*); BP, BP-treated group (*Bacillus pumilus* treated group).

## Data Availability

Data are not deposited in an official repository. The data belong to Southwest Minzu University and Animal Science Academy of Sichuan Province but can be made available by requesting them from the corresponding author.

## Supplementary Materials

The following are available online at https://www.mdpi.com/2076-2615/10/9/1604/s1, File S1: Methods of rumen and caecum microbiota analyses; Figure S1: correlation analysis of genera abundance information among different microbial Communities.
