# Supplementary material for: Effects of Bacillus amyloliquefaciens and Bacillus pumilus on Rumen and Intestine Morphology and Microbiota in Weanling Jintang Black Goat
Source: Animals (Basel). 2020 Sep 9;10(9):1604. doi: 10.3390/ani10091604 (PMC7552323; doi:10.3390/ani10091604)
Supplement: Supplementary file 1 [file animals-10-01604-s001.zip › animals-884361-supplementary/supplementary.docx]

Supplementary Materials: Effects of *Bacillus Amyloliquefaciens* and *Bacillus Pumilus* on Rumen and Intestine Morphology and Microbiota in Weanling Jintang Black Goat

Nanchi Zhang ^1^, Li Wang ^1,^* and Yong Wei ^2,^*

^1^ Key Laboratory of Qinghai-Tibetan Plateau Animal Genetic Resource Reservation and Utilization, Ministry of Education and Sichuan Province, Southwest Minzu University, Chengdu 610041, China; zhangnanchi@126.com

^2^ Animal Genetics and Breeding Key Laboratory of Sichuan Province, Animal Science Academy of Sichuan Province, Chengdu 610066, China

***** Correspondence: qinxin916@aliyun.com (L.W.); veishangyan@163.com (Y.W.); Fax: +86-28-85522310 (L.W.)


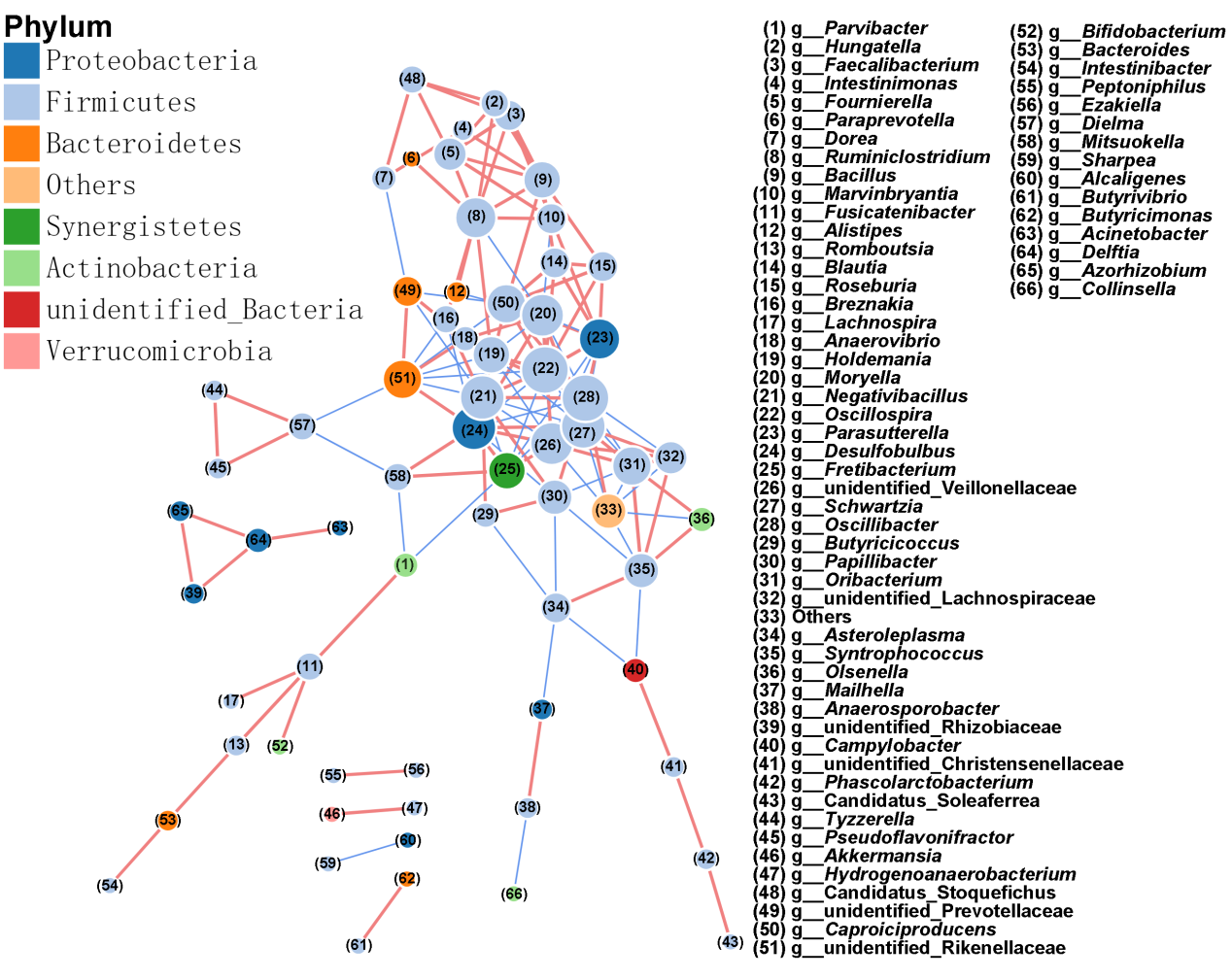


Figure S1. Correlation analysis of genera abundance information among different microbial Communities. Node size indicates the abundance, and color corresponds to phylum taxonomic classification. The width of lines were positively correlated with absolute values of correlation coefficients. The red line indicates positive correlation, and the blue line indicates negative correlation.
